# Autonomous Dam Surveillance Robot System Based on Multi-Sensor Fusion †

**DOI:** 10.3390/s20041097

**Published:** 2020-02-17

**Authors:** Chao Zhang, Quanzhong Zhan, Qi Wang, Haichao Wu, Ting He, Yi An

**Affiliations:** 1 Information Center of the Ministry of water resources of the P.R.C, Beijing 100053, China; zhangchao@mwr.gov.cn (C.Z.); zcqcz@mwr.gov.cn (Q.Z.); heting@mwr.gov.cn (T.H.); 2 Beijing Tritalent Intelligence Technology Co., Ltd., Beijing 100078, China; 3 School of Control Science and Engineering, Dalian university of technology, Dalian 116024, China

**Keywords:** water conversancy robot, dam surveillance, autonomous navigation, fusion localization

## Abstract

Dams are important engineering facilities in the water conservancy industry. They have many functions, such as flood control, electric power generation, irrigation, water supply, shipping, etc. Therefore, their long-term safety is crucial to operational stability. Because of the complexity of the dam environment, robots with various kinds of sensors are a good choice to replace humans to perform a surveillance job. In this paper, an autonomous system design is proposed for dam ground surveillance robots, which includes general solution, electromechanical layout, sensors scheme, and navigation method. A strong and agile skid-steered mobile robot body platform is designed and created, which can be controlled accurately based on an MCU and an onboard IMU. A novel low-cost LiDAR is adopted for odometry estimation. To realize more robust localization results, two Kalman filter loops are used with the robot kinematic model to fuse wheel encoder, IMU, LiDAR odometry, and a low-cost GNSS receiver data. Besides, a recognition network based on YOLO v3 is deployed to realize real-time recognition of cracks and people during surveillance. As a system, by connecting the robot, the cloud server and the users with IOT technology, the proposed solution could be more robust and practical.

## 1. Introduction

In the water conservancy industry, there are many fundamental engineering facilities, such as dams, water and soil conservation, water transfer project, shipping project, water supply project, hydraulic power plants, irrigation facilities, etc. Big water dams are the most comprehensive facilities. They always have many functions, such as flood control, electric power generation, irrigation, water supply, shipping, etc. [1]. Therefore, the safety of big dams is crucial to cities and people around. 

In the past, staff must check the environment, structure, and electromechanical facilities of dams regularly every day. This is a necessary way of keeping the dam running safely. However, it cannot realize all-weather all-day monitor and sometimes staffs are at risk since most dams are constructed in remote rural areas. Nowadays, with the great improvement of robot technology, robots have been used in public safety, security check, disaster rescue, and high-voltage lines inspection [2,3,4,5]. Many researchers and engineers are also paying attention to utilize underwater robots, unmanned aerial vehicles, unmanned surface vehicles for dams’ surveillance and inspection. 

However, applications of ground mobile robots are very rare. It is not easy for a ground robot to do the dam surveillance job autonomously. Firstly, the working places for most dams are off-road terrains, which needs a small, powerful, and agile robot mechanics design. Secondly, the GNSS satellite signal cannot fully cover all working area. The robot is difficult to continue path planning without accurate position information. Besides, to make a comprehensive check for a dam, the robot needs carry kinds of sensors. The robot must be capable of real-time data analyzing. In addition to the above, the robot system has to be low cost and robust.

In the autonomous robot area, simultaneous localization and mapping (SLAM) technology has been applied in many practical robot applications, especially laser SLAM. Researchers have proposed many laser-SLAM algorithms to build 2D/3D maps and realize localization [6,7]. Visual odometer (VO) is another hot topic in this area [8,9]. Cameras are relatively cheap and visual algorithms could show better performance in some environments. Many path planning methods are also being proposed to solve the obstacle avoiding problem [10,11]. The navigation technology now is ready for mobile robot design. Developing an autonomous dam surveillance robot system presents a wide variety of challenges. The robot should move on all kinds of ground surfaces and sometimes should move on narrow spaces. GNSS signal cannot cover all the work areas and it is difficult to acquire an accurate position. Online real-time inspection should be applied to the robot and the inspection algorithms should work with small computing resources.

This paper presents a general system for the mobile robot, which includes the robot, the cloud server, and terminals. The system can watch surveillance jobs status and can control the robot remotely when an emergency. As the robot body itself, we design a small powerful wheeled skid-steering mobile platform. Compared to alternative wheel configurations such as Ackerman or axle-articulated, the skid-steering platform shows two major advantages. First, it is simple and robust in mechanical terms. Second, it provides better maneuverability, including zero-radius turning. After analyzing the kinematic model of the skid-steered robot, we can estimate wheel odometry well with an onboard IMU (inertial measurement unit).

In this paper, a low-cost LiDAR sensor is used for laser odometry. This Livox MID40 LiDAR is a high-performance low-cost LiDAR sensor easily available. However, the LiDAR only has a 38.4° FOV and has a novel non-repeat scan mode, which differs a lot with common laser rangefinders. The method LiDAR odometry and mapping (LOAM) is adopted here for odometry estimation from its point cloud data. The method here avoids high computing burden since we only use the data for odometry calculation, not for real-time mapping. Besides, the robot was equipped with a GNSS receiver and an IMU. Therefore, we have position data from different sources: the LiDAR odometry, the longitude and latitude data, the wheel odometry and IMU measured data. To build a robust and fast localization system, we designed two extended Kalman filter (EKF) nodes. The first EKF node fuses IMU data, wheel odometry, LiDAR odometry to get robot transform in the inertial frame. By running it on the robot STM32 MCU board, we can ensure the fusion has real-time performance with high frequency. The second fuses results of first node, IMU data, longitude and latitude data to get robot transform in the world frame. The second node is running on the upper PC. The longitude and latitude data would be the observer position when the GNSS signal is good. After the position is acquired, we use a dynamic window approach (DWA) algorithm to do motion planning.

The paper is organized as follows. Some related work on robot control, fusion localization, object detection methods is summarized in Section 2. The general robot design is shown in Section 3. The mechanical design and control of the mobile robot can be seen in Section 4. All mounted sensors would be introduced in Section 5. Localization solution and some practical results could be found in Section 6. The online detection method is described in Section 7. Finally, a short conclusion is given in Section 8.

## 2. Related Work

To solve the dam surveillance and inspecting the problem, many robotics and sensor technologies have been deployed. A GPS-based surveillance robot is proposed in [12] to monitor the exterior deformation of the dam. A mechanically scanned imaging sonar (MSIS) is used for locating the underwater robot relative position for dam wall surveillance [13]. Many researchers utilized UAVs for dam image collecting and modeling, which is low-cost and convenient [14,15].

The wheeled skid-steering mobile robot is agile and strong, but its kinematics is complex since the drift always occurs and difficult to measure [16]. In [16], an IMU-based EKF fusion method is also proposed to improve the wheel odometry. To improve real-time motion control and dead-reckoning, a method to experimentally obtain an optimized kinematic model of the robot is presented in [17]. A detail model analysis and a back-stepping controller design are proposed in [18], simulation results for trajectory tracking are good. To find the correct model parameters [19], designed analysis and experimental kinematic scheme for skid-steering wheeled robot based-on a laser scanner sensor. The practical experiments show improvement in dead-reckoning performance.

A real-time kinematic (RTK) GPS sensor can help the robot to get an accurate position. It is commonly used in wide-open space [20]. Laser SLAM is now a relatively mature way to do mapping and localization (LiDAR odometry and mapping). LOAM proposed in [21] is one of the best LiDAR SLAM methods. The LOAM was running on the Velodyne Puck LiDAR which has 16 laser lines. Recently, a novel version of LOAM is proposed in [22], which is running on a low-cost novel Livox LiDAR and shows good performance in experiments. For land robots, dead reckoning is another reliable source of odometry. A robust AHRS could improve the wheel dead reckoning well [23].

Localization is one of the most important elements in the robot system. When the GPS/GNSS signal is too weak to use, a framework combines keyframe-based visual-inertial odometry with novel geometric image-based localization is built to provide a real-time estimate of a UAV’s pose. Weak GPS feeds are used as a weak prior for suggesting loop closures [24]. A Monte Carlo Localization system that fuses wheel and visual odometry for the prediction are designed for sewer inspection robots. The update step takes into account the network topology and other active methods. The system works well in practical experiments [25]. To address existing positioning systems do not perform well in urban canyons, tunnels, and indoor parking lots for driverless cars, an EKF based multi-sensor positioning system that combines a GPS sensor, a camera, and in-vehicle sensors assisted by kinematic and dynamic vehicle models [26]. Among all the localization system, EKF is widely used and shows good performances [27,28].

For dam surveillance, people and dam crack detection are important to ensure people and dam safety. Vision-based approaches to detect, track and identify people on a mobile robot in real-time has been used for many years. Thermal and grey images are successfully used to do the people recognition job on a mobile security robot [29]. Deep Learning shows great potential for crack damage detection. A deep architecture of convolutional neural networks (CNNs) method is proposed in [30] to find concrete cracks for civil infrastructures.

Therefore, a wheeled skid-steering mobile robot is built for the dam surveillance, which carries an onboard IMU, wheel encoders, a Livox LiDAR sensor, a GNSS antenna, a monocular industrial camera, and a customized MCU controller. We proposed the robot platform controller design, odometry methods, fusion localization, crack detection network to make the whole system low-cost and robust.

## 3. General Design

In dam surveillance applications, there are three main modules, which are the robot itself, the cloud server and the front-end terminals. The robot is responsible for moving in the workplace, collecting monitor data and handling some online detection applications. The cloud server takes charge of data processing and transferring. The users can watch the robot status, do manual analysis, and control the robot remotely based on real-time video streaming when necessary.

**Figure 1:**
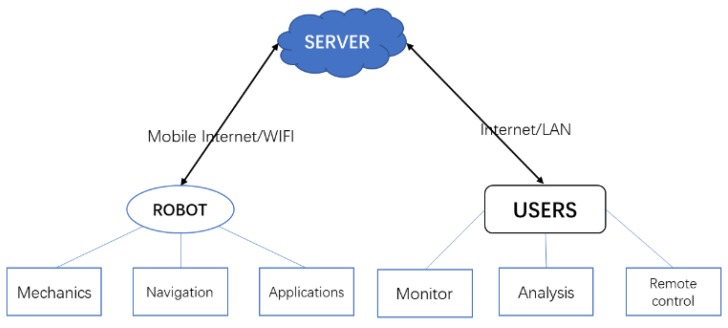
General design scheme for a dam surveillance robot.

The robot and the server are connected by mobile internet and WIFI. The server and the terminal users are connected by the Internet and LAN. The general structure diagram is shown in Figure 1. The robot itself and the cloud server are connected via message queuing telemetry transport protocol (MQTT). Robot navigation data can transfer to the server and the server can send job command and global navigation plan to the robot. This paper mainly focuses on the robot module.

**Figure 2:**
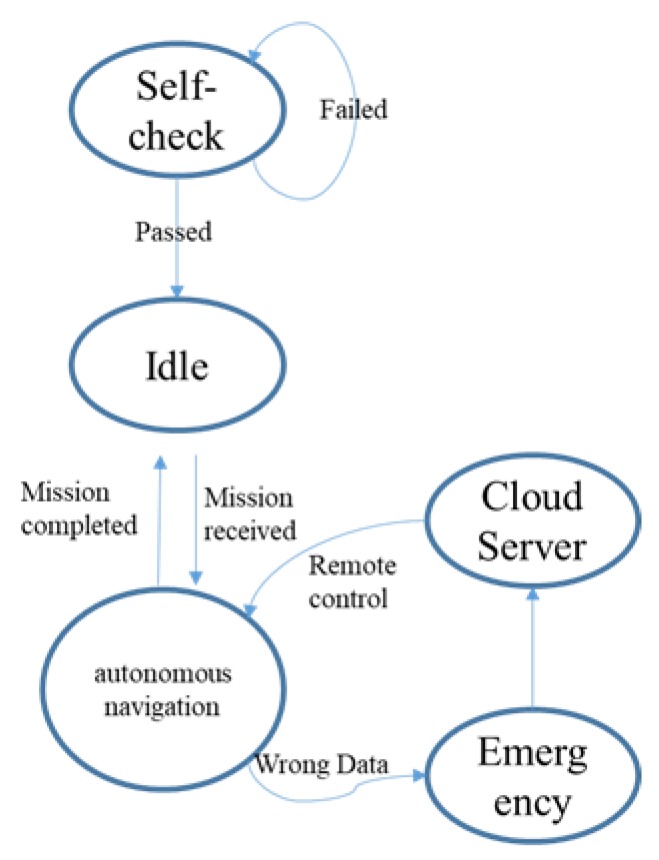
State machine logic structure.

A state machine is designed here for robot control, which includes self-check, autonomous navigation, remote control, idle, and emergency states. The robot would get into self-check state once the power is turned on. All sensors, motors, localization data, the high-level computer will then be checked. The robot would get into an idle state and wait for task command from the cloud server if the self-check is passed. Autonomous navigation would be triggered once the task and waypoints are received. The emergency state would be triggered when the robot is stuck or some sensors are running wrong. The logic of the state machine is shown in Figure 2.

## 4. Robot Platform

To realize high robustness and agile motion performance, the robot body is built with an all-aluminum chassis which about a total of 10 Kg weight. Two 200W DC brushless high torque motors are deployed to realize four-wheel drive. A 24 V lithium battery with 40 AH capacity is used as the power supply.

**Figure 3:**
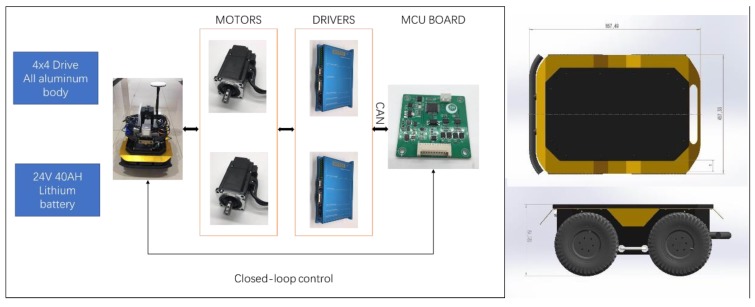
Mechanical layout and onboard control diagram.

All motors are controlled by a customized MCU board, which connects the motors and drivers by CAN bus. The maximal speed is 2 m/s; the maximum payload is 40 Kg; the maximum runtime is more than 4 hours. It is suitable for rugged all-terrain operation with four off-road tires. Figure 3 shows the body design.

**Figure 4:**
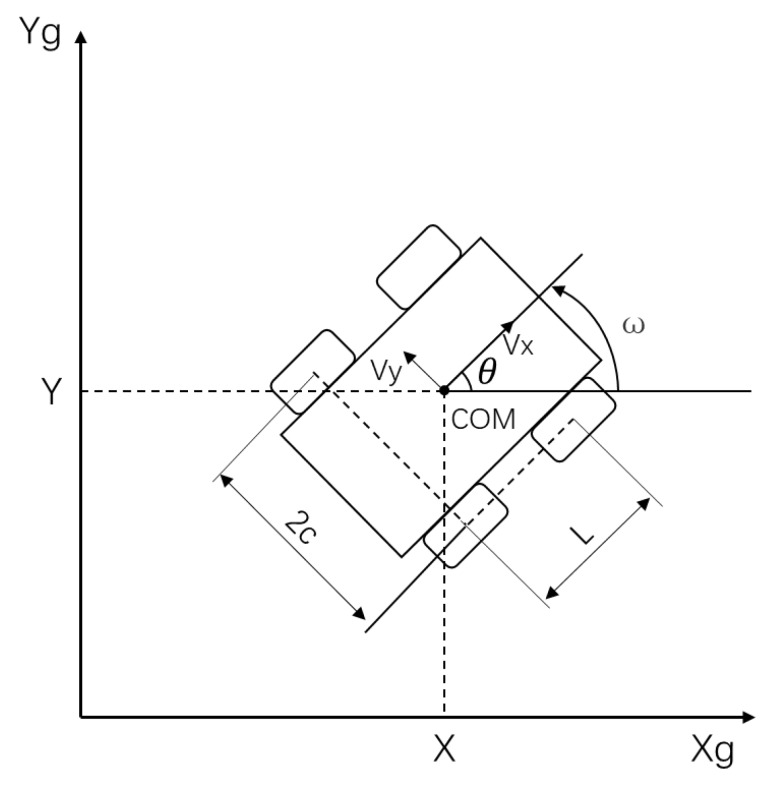
Kinematic model of the robot.

As shown in Figure 4, for the robot body, we will use the velocity and the angular velocity in the robot axis as state variables, i.e., ${\left[v_{x}\;w\right]}^{T}$. After the kinematic analysis of the robot, we can find the relationship between robot velocities and wheel speed.

(1) $$\left[\begin{array}{c} v_{x}\\ w \end{array}\right]=\;r\frac{\pi d}{60n}\left[\begin{array}{cc} \frac{1}{2} & \frac{1}{2}\\ -\frac{1}{2c} & -\frac{1}{2c} \end{array}\right]\left[\begin{array}{c} W_{l}\\ W_{r} \end{array}\right]$$

where *r* is so called effective radius of wheels, *n* is the reduction ratio, 2*c* is a spacing wheel track.

For the robot body, it will get velocity and angular velocity command ${\left[v_{x}^{d}\;w^{d}\right]}^{T}$ from upper PC. We should control the velocities to track the command. From (1), we have

(2) $$\{\begin{array}{c} W_{l}+W_{r}=\frac{2v_{x}^{d}}{r\frac{\pi d}{60n}}\\ -W_{l}+W_{r}=\frac{2w_{}^{d}c}{r\frac{\pi d}{60n}} \end{array}$$

**Figure 5:**
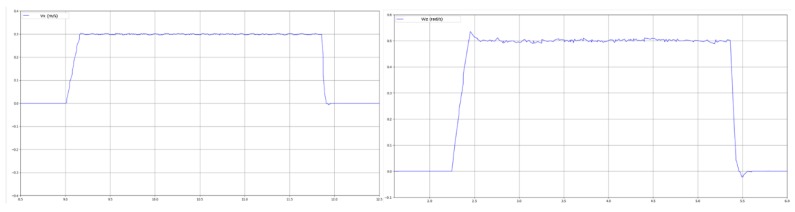
Practical response of the forward velocity and angular velocity.

From (2), we could calculate the desired wheel velocities easily, but the direct calculation does not work well since the model is a realistic model and the angular velocity always has a large lag. Therefore, we add an onboard IMU and use the following equation to set the wheel velocities.

(3) $$\{\begin{array}{c} v_{x}=v_{x}^{d}\\ w=k_{p}e_{w}+k_{i}\int^{\;}e_{w}+k_{d}{\dot{e}}_{w} \end{array}$$

where $k_{p},k_{i},k_{d}$ are constants for the simple PID controller, $e_{w}=w^{d}-w^{i}$,$\;w^{i}$ is the feedback from the onboard IMU. The practical response could be seen in Figure 5.

**Figure 6:**
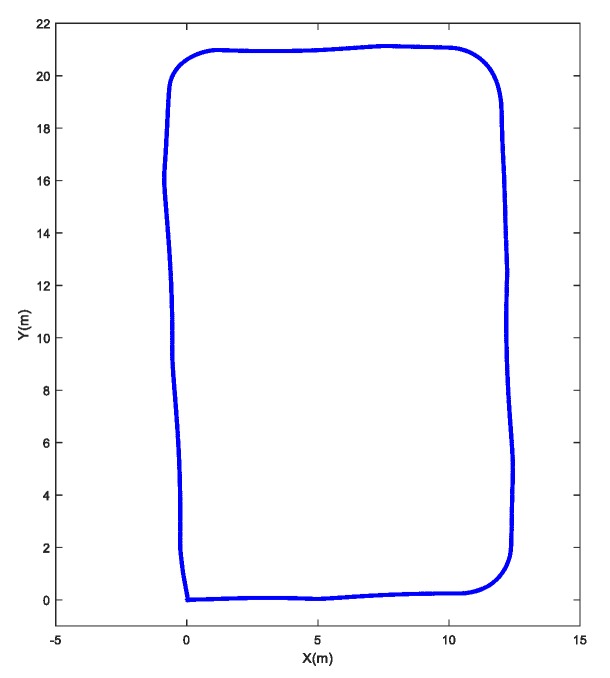
Dead reckoning results.

It is simple to calculate the wheel odometry in the inertial frame. We drive the robot moving a square and back to the origin, the dead reckoning result is shown in Figure 6 and final position value is [0.1229–0.2654 0.03] (should be [0 0 0]^T^). The whole distance is about 70 m and we did experiments 10 times, the origin RMSE (root mean square errors) is 0.3135.

(4) $$\{\begin{array}{c} \left[\begin{array}{c} \dot{X}\\ \dot{Y} \end{array}\right]=\left[\begin{array}{cc} \cos\theta & \sin\theta \\ \sin\theta & \cos\theta \end{array}\right]\left[\begin{array}{c} v_{x}\\ v_{y} \end{array}\right]\\ \dot{\theta =w^{i}}\\ v_{y}=-X_{ICR}\cdot w^{i} \end{array}Z$$

## 5. Sensors

**Figure 7:**
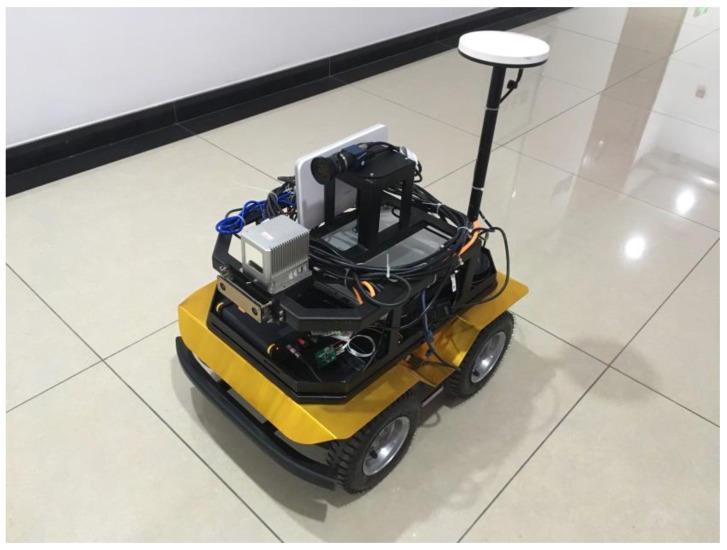
Experiment robot with sensors.

The experimental robot with sensors is shown in Figure 7. From the front to the back, there are 1 GNSS receiver, a stereo camera, a Livox LiDAR, a monocular camera, a 4G LTE wireless router, an industrial computer, a GNSS antenna. An onboard MCU controller with IMU is located inside the body box. Motion control is handled by the onboard MCU which also sends odometry data to the high-level industrial computer. The navigation is handled by the computer which is connected to the cloud server.

A navigation map is built mainly by the LiDAR and odometry data using the LOAM SLAM method. The map would be regarded as prior knowledge. The monocular camera in the middle is used to transfer video streaming and do the people and crack detection jobs. The GNSS receiver with antenna can acquire accurate position data in the wide-open area. On the other hand, the LiDAR’s point cloud and cameras’ depth data are designed to be used in obstacles perception as well in the future.

## 6. Localization and Navigation

**Figure 8:**
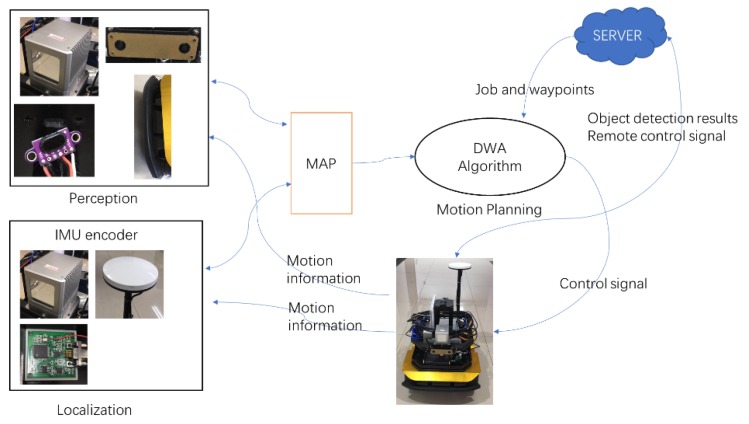
Navigation diagram.

The navigation includes perception, localization, path planning, and a remote controller. The whole diagram is depicted in Figure 8. The mature cost grid map is used for general environment representation. The filtered point cloud and depth data would be obstacles shown on the map.

The path planning method is divided into two parts here. The global waypoints are sent to the robot by the cloud server, which is pre-defined by different jobs. The local motion planning is achieved by the DWA algorithm on the grid map.

Localization is one of the most difficult problems for dam robots since the environment is changing and the robot has to move indoor and outdoor. Using one single sensor to acquire a positon is an impossible task. Many sensors would introduce fusion problem. Extended Kalman filter (EKF) is a good way to solve this problem and has been deployed in many robot localization applications.

A two EKF node structure is proposed here to build a robust localization system. The GNSS data is transformed based on the local origin and then input to EKF global node update process. The yaw angle from the LiDAR odometry is input to the EKF update process. The result of the EKF local node is input to the EKF global predict process. The LiDAR odometry data are input to the EKF local update process. The wheel odometry data and the IMU measurement is input to the EKF local predict process. It is noted here that the local EKF node is running on the embedded MCU at 200Hz and it handles in 3D space with 16 states.

**Figure 9:**
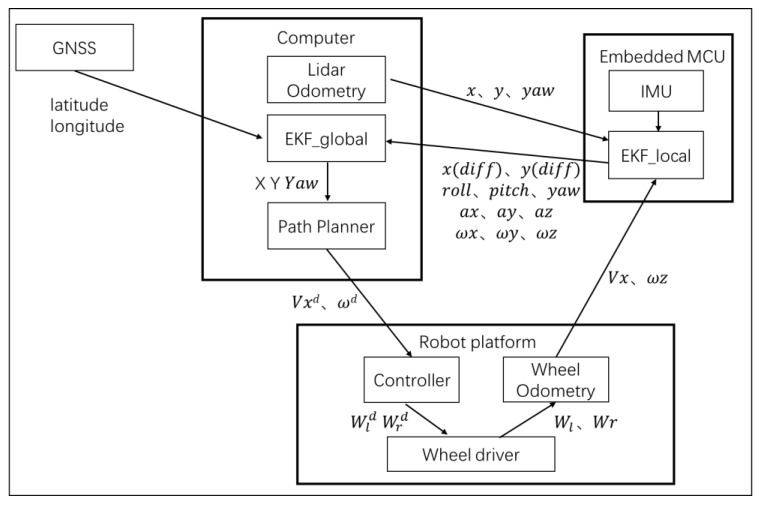
Fusion localization structure diagram.

It is important to get the 3D pose information for the rubber ground surface. However, the global EKF node is running at 30Hz on the computer and it only produces in 2D space. The robot position and orientation in the map are acquired with the EKF global updating. The fusion localization structure diagram is shown in Figure 9. Where $W_{l}^{d},W_{l}^{d}$ are the wheel velocity command, $W_{l}^{},W_{l}^{}$ are the feedback wheel velocity from encoders, $V_{x}^{d},\omega_{}^{d}$ are the velocity command generated from motion planning, $V_{x}^{},{\omega z}_{}^{}$ are the wheel odometry outputs, x,y,yaw are the LiDAR odometry, X,Y,Yaw are the final global pose estimation result.

With the kinematic models and zero-velocity constraints, we are now ready to design a kinematic-model-based robot positioning scheme for the local EKF node. The general state update equation is

(5) $$x_{t}=Fx_{t-1}+Gu_{t}$$

where $x_{t}={\left[q_{0}\;q_{1}\;q_{2}\;q_{3}\;x\;y\;z\;V_{x}\;V_{y}\;V_{z}\;\delta \varphi_{bias}\;\delta \theta_{bias}\;\delta \psi_{bias}\;\delta V_{xbias}\;\delta V_{ybias}\;\delta V_{zbias}\right]}^{T}$ is the state variables, $q_{0}\;q_{1}\;q_{2}\;q_{3}$ are the quaternion, x y z are the position in the inertial frame, $V_{x}\;V_{y}\;V_{z}$ are the corresponding velocities, $\delta \varphi_{bias}\;\delta \theta_{bias}\;\delta \psi_{bias}$ are the rotation zero-offset, $\delta V_{xbias}\;\delta V_{ybias}\;\delta V_{zbias}$ are the acceleration zero-offset. We have the following detail state update equations.

(6) $$\left[\begin{array}{c} q_{0}\left(k+1\right)\\ q_{1}\left(k+1\right)\\ q_{2}\left(k+1\right)\\ q_{3}\left(k+1\right) \end{array}\right]=q(h)•\left[\begin{array}{c} q_{0}\left(k\right)\\ q_{1}\left(k\right)\\ q_{2}\left(k\right)\\ q_{3}\left(k\right) \end{array}\right]$$

(7) $$q(h)=\left[\begin{array}{c} 1\\ \frac{\delta \phi }{2}-\frac{\delta \phi {\;}_{bias}}{2}\\ \frac{\delta \theta }{2}-\frac{\delta \theta_{bias}\;}{2}\\ \frac{\delta \psi }{2}-\frac{\delta \psi_{bias}}{2} \end{array}\right]$$

where δϕ, δθ, δψ are the radian variations during dt.

(8) $$\left[\begin{array}{c} V_{x}\left(k+1\right)\\ V_{y}\left(k+1\right)\\ V_{z}\left(k+1\right) \end{array}\right]=\left[\begin{array}{c} V_{x}\left(k\right)\\ V_{y}\left(k\right)\\ V_{z}\left(k\right) \end{array}\right]+\left[\begin{array}{c} g_{x}\left(k\right)\\ g_{y}\left(k\right)\\ g_{z}\left(k\right) \end{array}\right]•dt+T_{b}^{n}\delta V$$

where $g_{x}\left(k\right)$$g_{y}\left(k\right)$$g_{z}\left(k\right)$ are the components of gravity.

(9) $$\delta V=\left[\begin{array}{c} \delta V_{x}\\ \delta V_{y}\\ \delta V_{z} \end{array}\right]-\left[\begin{array}{c} \delta V_{xbias}\\ \delta V_{ybias}\\ \delta V_{zbias} \end{array}\right]$$

where $\delta V_{xbias}$, $\delta V_{ybias}$, $\delta V_{zbias}$ are the velocity variations during dt.

(10) $$\left[\begin{array}{c} x\left(k+1\right)\\ y\left(k+1\right)\\ z\left(k+1\right) \end{array}\right]=\left[\begin{array}{c} x\left(k\right)\\ y\left(k\right)\\ z\left(k\right) \end{array}\right]+\left[\begin{array}{c} V_{x}\left(k\right)\\ V_{y}\left(k\right)\\ V_{z}\left(k\right) \end{array}\right]•dt$$

(11) $$\left[\begin{array}{c} \delta \phi_{bias}(k+1)\\ \delta \theta_{bias}(k+1)\\ \delta \psi_{bias}(k+1) \end{array}\right]=\left[\begin{array}{c} \delta \phi_{bias}(k)\\ \delta \theta_{bias}(k)\\ \delta \psi_{bias}(k) \end{array}\right]$$

(12) $$\left[\begin{array}{c} \delta V_{xbias}(k+1)\\ \delta V_{ybias}(k+1)\\ \delta V_{zbias}(k+1) \end{array}\right]=\left[\begin{array}{c} \delta V_{xbias}(k)\\ \delta V_{ybias}(k)\\ \delta V_{zbias}(k) \end{array}\right]$$

The general time update equation is

(13) $$P_{k}=F_{k-1}P_{k-1}F^{T}{}_{k-1}+G_{k-1}Q_{k-1}G^{T}{}_{k-1}+Q_{s}$$

where, $P_{k}$ is the covariance matrix, $F_{k}={\left(\frac{\partial f}{\partial x}\right)}_{k}$ is state Jacobian, $G_{k}={\left(\frac{\partial f}{\partial u}\right)}_{k}$ is control input Jacobian, $Q_{k-1}$ is sensor process noise, $Q_{s}$ is an additional process noise. The state transform matrix have

(14) $$F_{k}=\left[\begin{array}{cccccccccccccccc} 1 & F_{0,1} & F_{0,2} & F_{0,3} & 0 & 0 & 0 & 0 & 0 & 0 & F_{0,10} & F_{0,11} & F_{0,12} & 0 & 0 & 0\\ F_{1,0} & 1 & F_{1,2} & F_{1,3} & 0 & 0 & 0 & 0 & 0 & 0 & F_{1,10} & F_{1,11} & F_{1,12} & 0 & 0 & 0\\ F_{2,0} & F_{2,1} & 1 & F_{2,3} & 0 & 0 & 0 & 0 & 0 & 0 & F_{2,10} & F_{2,11} & F_{2,12} & 0 & 0 & 0\\ F_{3,0} & F_{3,1} & F_{3,2} & 1 & 0 & 0 & 0 & 0 & 0 & 0 & F_{3,10} & F_{3,11} & F_{3,12} & 0 & 0 & 0\\ F_{4,0} & F_{4,1} & F_{4,2} & F_{4,3} & 1 & 0 & 0 & 0 & 0 & 0 & 0 & 0 & 0 & F_{4,13} & F_{4,14} & F_{4,15}\\ F_{5,0} & F_{5,1} & F_{5,2} & F_{5,3} & 0 & 1 & 0 & 0 & 0 & 0 & 0 & 0 & 0 & F_{5,13} & F_{5,14} & F_{5,15}\\ F_{6,0} & F_{6,1} & F_{6,2} & F_{6,3} & 0 & 0 & 1 & 0 & 0 & 0 & 0 & 0 & 0 & F_{6,13} & F_{6,14} & F_{6,15}\\ 0 & 0 & 0 & 0 & dt & 0 & 0 & 1 & 0 & 0 & 0 & 0 & 0 & 0 & 0 & 0\\ 0 & 0 & 0 & 0 & 0 & dt & 0 & 0 & 1 & 0 & 0 & 0 & 0 & 0 & 0 & 0\\ 0 & 0 & 0 & 0 & 0 & 0 & dt & 0 & 0 & 1 & 0 & 0 & 0 & 0 & 0 & 0\\ 0 & 0 & 0 & 0 & 0 & 0 & 0 & 0 & 0 & 0 & 1 & 0 & 0 & 0 & 0 & 0\\ 0 & 0 & 0 & 0 & 0 & 0 & 0 & 0 & 0 & 0 & 0 & 1 & 0 & 0 & 0 & 0\\ 0 & 0 & 0 & 0 & 0 & 0 & 0 & 0 & 0 & 0 & 0 & 0 & 1 & 0 & 0 & 0\\ 0 & 0 & 0 & 0 & 0 & 0 & 0 & 0 & 0 & 0 & 0 & 0 & 0 & 1 & 0 & 0\\ 0 & 0 & 0 & 0 & 0 & 0 & 0 & 0 & 0 & 0 & 0 & 0 & 0 & 0 & 1 & 0\\ 0 & 0 & 0 & 0 & 0 & 0 & 0 & 0 & 0 & 0 & 0 & 0 & 0 & 0 & 0 & 1 \end{array}\right]$$

Therefore, we can acquire

(15) $$F_{0,1}=\frac{\partial q_{0}(t+\Delta t)}{\partial q_{1}}=\delta \phi {\;}_{bias}/2-\delta \phi /2;$$

(16) $$F_{0,2}=\frac{\partial q_{0}(t+\Delta t)}{\partial q_{2}}=\delta \theta_{bias}/2-\delta \theta /2;$$

(17) $$F_{0,3}=\frac{\partial q_{0}(t+\Delta t)}{\partial q_{3}}=\delta \psi_{bias}/2-\delta \psi /2;$$

(18) $$F_{0,10}=\frac{\partial q_{0}(t+\Delta t)}{\partial \delta \phi {\;}_{bias}}=\frac{q_{1}}{2};$$

(19) $$F_{0,11}=\frac{\partial q_{0}(t+\Delta t)}{\partial \delta \theta_{bias}}=\frac{q_{2}}{2};$$

(20) $$F_{0,12}=\frac{\partial q_{0}(t+\Delta t)}{\partial \delta \psi_{bias}}=\frac{q_{3}}{2};$$

(21) $$F_{1,0}=\frac{\partial q_{1}(t+\Delta t)}{\partial q_{0}}=\delta \phi /2-\delta \phi {\;}_{bias}/2;$$

(22) $$F_{1,2}=\frac{\partial q_{1}(t+\Delta t)}{\partial q_{2}}=\delta \psi /2-\delta \psi_{bias}/2;$$

(23) $$F_{1,3}=\frac{\partial q_{1}(t+\Delta t)}{\partial q_{3}}=\delta \theta_{bias}/2-\delta \theta /2;$$

(24) $$F_{1,10}=\frac{\partial q_{1}(t+\Delta t)}{\partial \delta \phi {\;}_{bias}}=-\frac{q_{0}}{2}$$

(25) $$F_{1,11}=\frac{\partial q_{1}(t+\Delta t)}{\partial \delta \theta_{bias}}=\frac{q_{3}}{2};$$

(26) $$F_{1,12}=\frac{\partial q_{1}(t+\Delta t)}{\partial \delta \psi_{bias}}=-\frac{q_{2}}{2};$$

(27) $$F_{2,0}=\frac{\partial q_{2}(t+\Delta t)}{\partial q_{0}}=\delta \theta /2-\delta \theta {\;}_{bias}/2;$$

(28) $$F_{2,1}=\frac{\partial q_{2}(t+\Delta t)}{\partial q_{1}}=\delta \psi_{bias}/2-\delta \psi /2;$$

(29) $$F_{2,3}=\frac{\partial q_{2}(t+\Delta t)}{\partial q_{3}}=\delta \phi /2-\delta \phi_{bias}/2;$$

(30) $$F_{2,10}=\frac{\partial q_{2}(t+\Delta t)}{\partial \delta \phi {\;}_{bias}}=-\frac{q_{3}}{2};$$

(31) $$F_{2,11}=\frac{\partial q_{2}(t+\Delta t)}{\partial \delta \theta_{bias}}=-\frac{q_{0}}{2};$$

(32) $$F_{2,12}=\frac{\partial q_{2}(t+\Delta t)}{\partial \delta \psi_{bias}}=\frac{q_{1}}{2};$$

(33) $$F_{3,0}=\frac{\partial q_{3}(t+\Delta t)}{\partial q_{0}}=\delta \psi /2-\delta \psi_{bias}/2;$$

(34) $$F_{3,1}=\frac{\partial q_{3}(t+\Delta t)}{\partial q_{1}}=\delta \theta /2-\delta \theta_{bias}/2;$$

(35) $$F_{3,2}=\frac{\partial q_{3}(t+\Delta t)}{\partial q_{2}}=\delta \phi {\;}_{bias}/2-\delta \phi /2;$$

(36) $$F_{3,10}=\frac{\partial q_{3}(t+\Delta t)}{\partial \delta \phi {\;}_{bias}}=\frac{q_{2}}{2};$$

(37) $$F_{3,11}=\frac{\partial q_{3}(t+\Delta t)}{\partial \delta \theta_{bias}}=-\frac{q_{1}}{2};$$

(38) $$F_{3,12}=\frac{\partial q_{3}(t+\Delta t)}{\partial \delta \psi_{bias}}=-\frac{q_{0}}{2};$$

(39) $$F_{4,0}=\frac{\partial v_{x}(t+\Delta t)}{\partial q_{0}}=2*q0*(\delta V_{x}\;-\;\delta V_{xbias})\;-\;2*q3*(\delta V_{y}\;-\;\delta V_{ybias})\;+\;2*q2*(\delta V_{z}\;-\;\delta V_{zbias});$$

(40) $$F_{4,1}=\frac{\partial v_{x}(t+\Delta t)}{\partial q_{1}}=\;2*q1*(\delta V_{x}\;-\;\delta V_{xbias})\;+\;2*q2*(\delta V_{y}\;-\;\delta V_{ybias})\;+\;2*q3*(\delta V_{z}\;-\;\delta V_{zbias});$$

(41) $$F_{4,2}=\frac{\partial v_{x}(t+\Delta t)}{\partial q_{2}}=\;2*q1*(\delta V_{y}\;-\;\delta V_{ybias})\;-\;2*q2*(\delta V_{x}\;-\;\delta V_{xbias})\;+\;2*q0*(\delta V_{z}\;-\;\delta V_{zbias});$$

(42) $$F_{4,3}=\frac{\partial v_{x}(t+\Delta t)}{\partial q_{3}}=2*q1*(\delta V_{z}\;-\;\delta V_{zbias})\;-\;2*q0*(\delta V_{y}\;-\;\delta V_{ybias})\;-\;2*q3*(\delta V_{x}\;-\;\delta V_{xbias});$$

(43) $$F_{4,13}=\frac{\partial v_{x}(t+\Delta t)}{\partial \delta V_{xbias}}=-q0^{2}-q1^{2}+q2^{2}+q3^{2}$$

(44) $$F_{4,14}=\frac{\partial v_{x}(t+\Delta t)}{\partial \delta V_{ybias}}=2*q0*q3\;-\;2*q1*q2$$

(45) $$F_{4,15}=\frac{\partial v_{x}(t+\Delta t)}{\partial \delta V_{zbias}}=-2(q_{0}*q_{2}+q_{1}*q_{3});$$

(46) $$F_{5,0}=\frac{\partial v_{y}(t+\Delta t)}{\partial q_{0}}=2*q3*(\delta V_{x}\;-\;\delta V_{xbias})\;+\;2*q0*(\delta V_{y}\;-\;\delta V_{ybias})\;-\;2*q1*(\delta V_{z}\;-\;\delta V_{zbias});$$

(47) $$F_{5,1}=\frac{\partial v_{y}(t+\Delta t)}{\partial q_{1}}=2*q2*(\delta V_{x}\;-\;\delta V_{xbias})\;-\;2*q1*(\delta V_{y}\;-\;\delta V_{ybias})\;-\;2*q0*(\delta V_{z}\;-\;\delta V_{zbias});$$

(48) $$F_{5,2}=\frac{\partial v_{y}(t+\Delta t)}{\partial q_{2}}=2*q1*(\delta V_{x}\;-\;\delta V_{xbias})\;+\;2*q2*(\delta V_{y}\;-\;\delta V_{ybias})\;+\;2*q3*(\delta V_{z}\;-\;\delta V_{zbias});$$

(49) $$F_{5,3}=\frac{\partial v_{y}(t+\Delta t)}{\partial q_{3}}=2*q0*(\delta V_{x}\;-\;\delta V_{xbias})\;-\;2*q3*(\delta V_{y}\;-\;\delta V_{ybias})\;+\;2*q2*(\delta V_{z}\;-\;\delta V_{zbias});$$

(50) $$F_{5,13}=\frac{\partial v_{y}(t+\Delta t)}{\partial \delta V_{xbias}}=\;-\;2*q0*q3\;-\;2*q1*q2$$

(51) $$F_{5,14}=\frac{\partial v_{y}(t+\Delta t)}{\partial \delta V_{ybias}}=-q0^{2}+q1^{2}-q2^{2}+q3^{2}$$

(52) $$F_{5,15}=\frac{\partial v_{y}(t+\Delta t)}{\partial \delta V_{zbias}}=2(q_{0}*q_{1}-q_{2}*q_{3});$$

(53) $$F_{6,0}=\frac{\partial v_{z}(t+\Delta t)}{\partial q_{0}}=2*q1*(\delta V_{y}\;-\;\delta V_{ybias})\;-\;2*q2*(\delta V_{x}\;-\;\delta V_{xbias})\;+\;2*q0*(\delta V_{z}\;-\;\delta V_{zbias});$$

(54) $$F_{6,1}=\frac{\partial v_{z}(t+\Delta t)}{\partial q_{1}}=\;2*q3*(\delta V_{x}\;-\;\delta V_{xbias})\;+\;2*q0*(\delta V_{y}\;-\;\delta V_{ybias})\;-\;2*q1*(\delta V_{z}\;-\;\delta V_{zbias});$$

(55) $$F_{6,2}=\frac{\partial v_{z}(t+\Delta t)}{\partial q_{2}}=\;2*q3*(\delta V_{y}\;-\;\delta V_{ybias})\;-\;2*q0*(\delta V_{x}\;-\;\delta V_{xbias})\;-\;2*q2*(\delta V_{z}\;-\;\delta V_{zbias});$$

(56) $$F_{6,3}=\frac{\partial v_{z}(t+\Delta t)}{\partial q_{3}}=2*q1*(\delta V_{x}\;-\;\delta V_{xbias})\;+\;2*q2*(\delta V_{y}\;-\;\delta V_{ybias})\;+\;2*q3*(\delta V_{z}\;-\;\delta V_{zbias});$$

(57) $$F_{6,13}=\frac{\partial v_{z}(t+\Delta t)}{\partial \delta V_{xbias}}=2*q0*q2\;-\;2*q1*q3$$

(58) $$F_{6,14}=\frac{\partial v_{z}(t+\Delta t)}{\partial \delta V_{ybias}}=-\;2*q0*q1\;-\;2*q2*q3$$

(59) $$F_{6,15}=\frac{\partial v_{z}(t+\Delta t)}{\partial \delta V_{zbias}}=-q0^{2}+q1^{2}+q2^{2}-q3^{2}$$

The control input Jacobian matrix is

(60) $$G_{k}=\left[\begin{array}{cccccc} -\frac{q1}{2} & -\frac{q2}{2} & -\frac{q3}{2} & 0 & 0 & 0\\ \mathrm{SG}(1) & -\frac{q3}{2} & \frac{q2}{2} & 0 & 0 & 0\\ \frac{q3}{2} & \mathrm{SG}(1) & -\frac{q1}{2} & 0 & 0 & 0\\ -\frac{q2}{2} & \frac{q1}{2} & \mathrm{SG}(1) & 0 & 0 & 0\\ 0 & 0 & 0 & \mathrm{SG}(4)\;-\;\mathrm{SG}(3)\;-\;\mathrm{SG}(2)\;+\;\mathrm{SG}(5) & \mathrm{SG}(8)\;-\;2*q0*q3 & \mathrm{SG}(7)\;+\;2*q0*q2\\ 0 & 0 & 0 & \mathrm{SG}(8)\;+\;2*q0*q3 & \mathrm{SG}(3)\;-\;\mathrm{SG}(2)\;-\;\mathrm{SG}(4)\;+\;\mathrm{SG}(5) & \mathrm{SG}(6)\;-\;2*q0*q1\\ 0 & 0 & 0 & \mathrm{SG}(7)\;-\;2*q0*q2 & \mathrm{SG}(6)\;+\;2*q0*q1 & \mathrm{SG}(2)\;-\;\mathrm{SG}(3)\;-\;\mathrm{SG}(4)\;+\;\mathrm{SG}(5)\\ 0 & 0 & 0 & 0 & 0 & 0\\ 0 & 0 & 0 & 0 & 0 & 0\\ 0 & 0 & 0 & 0 & 0 & 0\\ 0 & 0 & 0 & 0 & 0 & 0\\ 0 & 0 & 0 & 0 & 0 & 0\\ 0 & 0 & 0 & 0 & 0 & 0\\ 0 & 0 & 0 & 0 & 0 & 0\\ 0 & 0 & 0 & 0 & 0 & 0\\ 0 & 0 & 0 & 0 & 0 & 0\\ 0 & 0 & 0 & 0 & 0 & 0\\ 0 & 0 & 0 & 0 & 0 & 0\\ 0 & 0 & 0 & 0 & 0 & 0\\ 0 & 0 & 0 & 0 & 0 & 0\\ 0 & 0 & 0 & 0 & 0 & 0\\ 0 & 0 & 0 & 0 & 0 & 0\\ 0 & 0 & 0 & 0 & 0 & 0\\ 0 & 0 & 0 & 0 & 0 & 0 \end{array}\right]$$

where

(61) $$SG=\left[\begin{array}{c} \frac{q_{0}}{2}\\ q_{3}{}^{2}\\ q_{2}{}^{2}\\ q_{1}{}^{2}\\ q_{0}{}^{2}\\ {2*q}_{2}{*q}_{3}\\ {2*q}_{1}{*q}_{3}\\ {2*q}_{1}{*q}_{2} \end{array}\right]$$

The process noise matrix is

(62) $$Q_{k}=\left[\begin{array}{cccccccccccccccc} q_{0,0} & SQ(9) & SQ(8) & SQ(7) & 0 & 0 & 0 & 0 & 0 & 0 & 0 & 0 & 0 & 0 & 0 & 0\\ SQ(9) & q_{1,1} & SQ(6) & SQ(5) & 0 & 0 & 0 & 0 & 0 & 0 & 0 & 0 & 0 & 0 & 0 & 0\\ SQ(8) & SQ(6) & q_{2,2} & SQ(4) & 0 & 0 & 0 & 0 & 0 & 0 & 0 & 0 & 0 & 0 & 0 & 0\\ SQ(7) & SQ(5) & SQ(4) & q_{3,3} & 0 & 0 & 0 & 0 & 0 & 0 & 0 & 0 & 0 & 0 & 0 & 0\\ 0 & 0 & 0 & 0 & q_{4,4} & \mathrm{SQ}(3) & \mathrm{SQ}(2) & 0 & 0 & 0 & 0 & 0 & 0 & 0 & 0 & 0\\ 0 & 0 & 0 & 0 & \mathrm{SQ}(3) & q_{5,5} & \mathrm{SQ}(1) & 0 & 0 & 0 & 0 & 0 & 0 & 0 & 0 & 0\\ 0 & 0 & 0 & 0 & \mathrm{SQ}(2) & \mathrm{SQ}(1) & q_{6,6} & 0 & 0 & 0 & 0 & 0 & 0 & 0 & 0 & 0\\ 0 & 0 & 0 & 0 & 0 & 0 & 0 & 0 & 0 & 0 & 0 & 0 & 0 & 0 & 0 & 0\\ 0 & 0 & 0 & 0 & 0 & 0 & 0 & 0 & 0 & 0 & 0 & 0 & 0 & 0 & 0 & 0\\ 0 & 0 & 0 & 0 & 0 & 0 & 0 & 0 & 0 & 0 & 0 & 0 & 0 & 0 & 0 & 0\\ 0 & 0 & 0 & 0 & 0 & 0 & 0 & 0 & 0 & 0 & 0 & 0 & 0 & 0 & 0 & 0\\ 0 & 0 & 0 & 0 & 0 & 0 & 0 & 0 & 0 & 0 & 0 & 0 & 0 & 0 & 0 & 0\\ 0 & 0 & 0 & 0 & 0 & 0 & 0 & 0 & 0 & 0 & 0 & 0 & 0 & 0 & 0 & 0\\ 0 & 0 & 0 & 0 & 0 & 0 & 0 & 0 & 0 & 0 & 0 & 0 & 0 & 0 & 0 & 0\\ 0 & 0 & 0 & 0 & 0 & 0 & 0 & 0 & 0 & 0 & 0 & 0 & 0 & 0 & 0 & 0\\ 0 & 0 & 0 & 0 & 0 & 0 & 0 & 0 & 0 & 0 & 0 & 0 & 0 & 0 & 0 & 0 \end{array}\right]$$

where,

(63) $$SQ=\left[\begin{array}{c} \mathrm{dvzCov}*(\mathrm{SG}(6)\;-\;2*q0*q1)*(\mathrm{SG}(2)\;-\;\mathrm{SG}(3)\;-\;\mathrm{SG}(4)\;+\;\mathrm{SG}(5))\;-\;\mathrm{dvyCov}*(\mathrm{SG}(6)\;+\;2*q0*q1)*(\mathrm{SG}(2)\;-\;\mathrm{SG}(3)\;+\;\mathrm{SG}(4)\;-\;\mathrm{SG}(5))\;+\;\mathrm{dvxCov}*(\mathrm{SG}(7)\;-\;2*q0*q2)*(\mathrm{SG}(8)\;+\;2*q0*q3)\\ \;\mathrm{dvzCov}*(\mathrm{SG}(7)\;+\;2*q0*q2)*(\mathrm{SG}(2)\;-\;\mathrm{SG}(3)\;-\;\mathrm{SG}(4)\;+\;\mathrm{SG}(5))\;-\;\mathrm{dvxCov}*(\mathrm{SG}(7)\;-\;2*q0*q2)*(\mathrm{SG}(2)\;+\;\mathrm{SG}(3)\;-\;\mathrm{SG}(4)\;-\;\mathrm{SG}(5))\;+\;\mathrm{dvyCov}*(\mathrm{SG}(6)\;+\;2*q0*q1)*(\mathrm{SG}(8)\;-\;2*q0*q3)\\ \mathrm{dvzCov}*(\mathrm{SG}(6)\;-\;2*q0*q1)*(\mathrm{SG}(7)\;+\;2*q0*q2)\;-\;\mathrm{dvyCov}*(\mathrm{SG}(8)\;-\;2*q0*q3)*(\mathrm{SG}(2)\;-\;\mathrm{SG}(3)\;+\;\mathrm{SG}(4)\;-\;\mathrm{SG}(5))\;-\;\mathrm{dvxCov}*(\mathrm{SG}(8)\;+\;2*q0*q3)*(\mathrm{SG}(2)\;+\;\mathrm{SG}(3)\;-\;\mathrm{SG}(4)\;-\;\mathrm{SG}(5))\\ (\mathrm{dayCov}*q1*\mathrm{SG}(1))/2\;-\;(\mathrm{dazCov}*q1*\mathrm{SG}(1))/2\;-\;(\mathrm{daxCov}*q2*q3)/4\\ (\mathrm{dazCov}*q2*\mathrm{SG}(1))/2\;-\;(\mathrm{daxCov}*q2*\mathrm{SG}(1))/2\;-\;(\mathrm{dayCov}*q1*q3)/4\\ (\mathrm{daxCov}*q3*\mathrm{SG}(1))/2\;-\;(\mathrm{dayCov}*q3*\mathrm{SG}(1))/2\;-\;(\mathrm{dazCov}*q1*q2)/4\\ (\mathrm{daxCov}*q1*q2)/4\;-\;(\mathrm{dazCov}*q3*\mathrm{SG}(1))/2\;-\;(\mathrm{dayCov}*q1*q2)/4\\ (\mathrm{dazCov}*q1*q3)/4\;-\;(\mathrm{daxCov}*q1*q3)/4\;-\;(\mathrm{dayCov}*q2*\mathrm{SG}(1))/2\\ (\mathrm{dayCov}*q2*q3)/4\;-\;(\mathrm{daxCov}*q1*\mathrm{SG}(1))/2\;-\;(\mathrm{dazCov}*q2*q3)/4\\ SG{\left(1\right)}^{2}\\ q1^{2} \end{array}\right]$$

(64) $$\begin{array}{ll} q_{0,0}= & (dayCov*q2^{2})/4+(dazCov*q3^{2})/4+(daxCov*SQ(11))/4\\ q_{1,1}= & \left(dazCov*q2^{2})/4+(dayCov*q3^{2})/4+daxCov*SQ(10\right)\\ q_{2,2}= & (daxCov*q3^{2})/4+dayCov*SQ(10)+(dazCov*SQ(11))/4\\ q_{3,3}= & \left(daxCov*q2^{2})/4+(dayCov*SQ(11))/4+dazCov*SQ(10\right)\\ q_{4,4}= & dvyCov*{\left(SG(8)-2*q0*q3\right)}^{2}+dvzCov*{\left(SG(7)+2*q0*q2\right)}^{2}\\ & +dvxCov*{\left(SG(2)+SG(3)-SG(4)-SG(5)\right)}^{2}\\ q_{5,5}= & dvxCov*{\left(SG(8)+2*q0*q3\right)}^{2}+dvzCov*{\left(SG(6)-2*q0*q1\right)}^{2}\\ & +dvyCov*{\left(SG(2)-SG(3)+SG(4)-SG(5)\right)}^{2}\\ q_{6,6}= & dvxCov*{\left(SG(7)-2*q0*q2\right)}^{2}+dvyCov*{\left(SG(6)+2*q0*q1\right)}^{2}\\ & +dvzCov*{\left(SG(2)-SG(3)-SG(4)+SG(5)\right)}^{2} \end{array}$$

where, daxCov,dayCov,dazCov are the radius variance, and dvxCov, dvyCov, dvzCov are the velocities variance.

The Kalman feedback matrix is

(65) $$K_{k}=P_{k}^{-}H_{k}{\left[H_{k}P_{k}^{-}H_{k}^{T}+R_{k}\right]}^{-1}$$

where

(66) $$H_{k}={\left(\frac{\partial z_{p}}{\partial x}\right)}_{k}$$

The covariance update equation is

(67) $$P_{k}^{+}=\left[I-K_{k}H_{k}\right]P_{k}^{-}$$

Finally, we could get the state update equation

(68) $$x_{k}^{+}=x_{k}^{-}+K_{k}v$$

**Figure 10:**
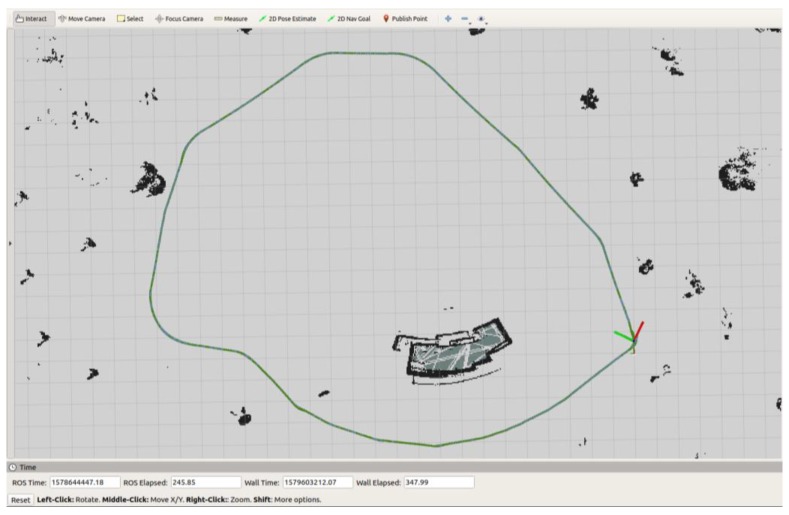
Practical localization performance on map.

The global EKF node is designed in the same method, the details are omitted here. The localization result in the practical environment can be found in Figure 10. The body coordinate represents the filtered pose of the robot now. The robot was running a circle in the practical environment. We can see that the localization performs well even sometimes the GNSS receiver is out of the lock and the result is ready for navigation. The localization and path planning could fail sometimes, even if so many sensors have been deployed. In this application, we designed a remote controller module. Users can control the robot remotely with real-time video streaming.

**Figure 11:**
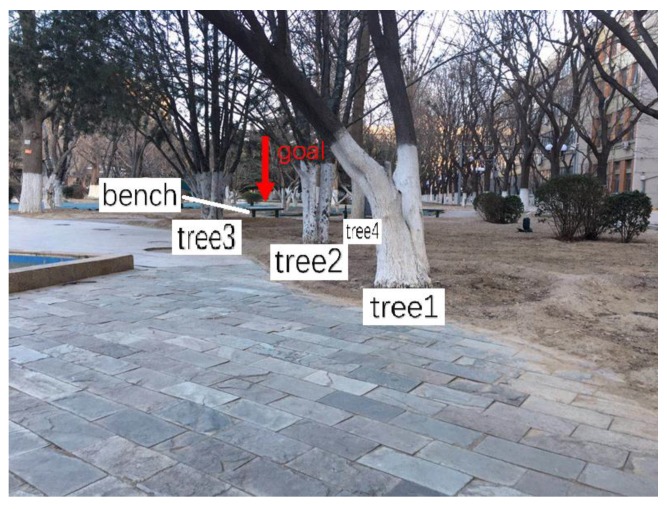
Experiment environment.

**Figure 12:**
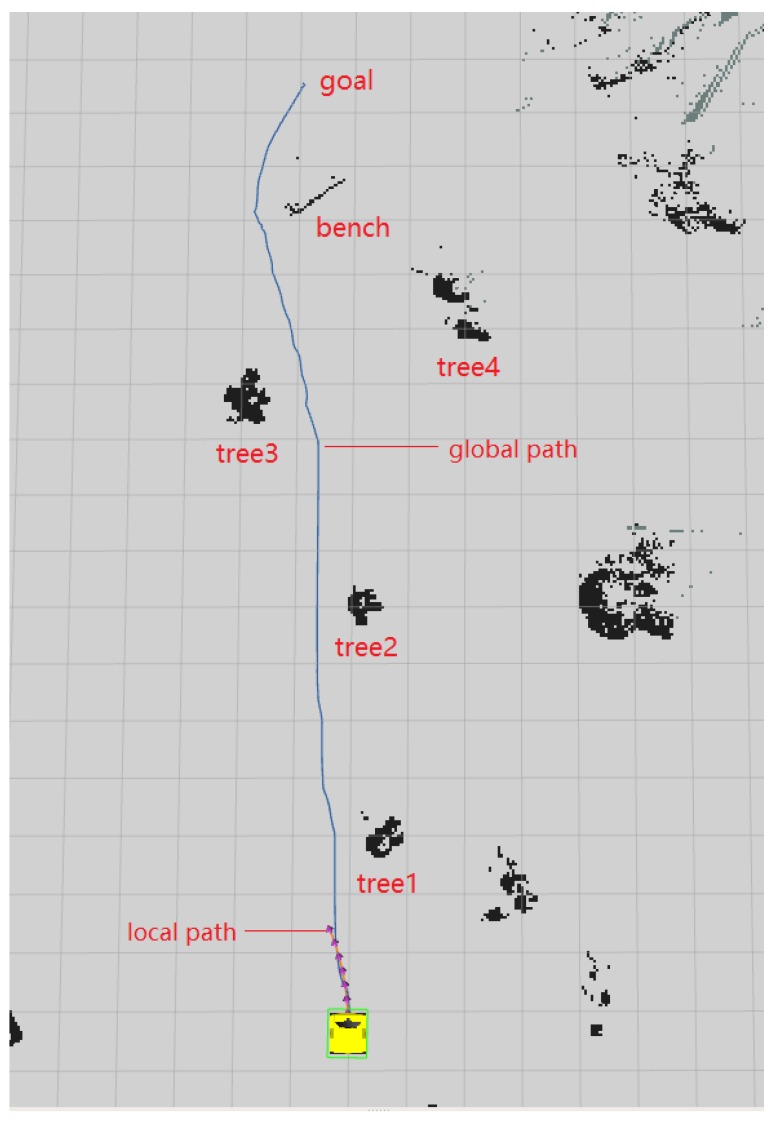
Navigation results.

The Figure 11 shows a picture of an experiment environment for a surveillance job, this picture is captured from the camera on the robot. In this environment, the road is narrow and the GNSS signal is weak because of many trees. However, the proposed robot system works well. The practical results could be seen in Figure 12. The goal and the blue line are the planned global path. The green line is the local path planning result. We can see the robot locate itself well and find where to go correctly.

## 7. Environment Inspection 

The dam crack identification and pedestrian detection are the key technologies related to the safety of the dam. We use the trained model of YOLO V3 [31] to recognize them. We use the dataset online to train the YOLO V3 algorithm and then recognize the dam crack. The YOLO V3 algorithm includes a darknet-53 module, eight DBL components, three convolutional layers, two upsampling layers, and two tensor concat layers. The darknet-53 includes a DBL component and five residual learning units res1, res2, res8, res8, and res4. 

The DBL contains a convolutional layer, a BN layer and a leaky ReLU. The convolutional parameters of the convolutional layer are the kernel size 3 × 3, stride 1, same padding and output channels 32. The res1, res2, res8, and res4 contain 1, 2, 8, and 4 basic units of the residual learning, respectively, and each basic unit of the residual learning contains two DBL and an identity map. The convolutional parameters of the first DBL are the kernel size 1 × 1, stride 1, and the output channels are equal to the number of basic units of the residual learning. The convolutional parameters of the second DBL are the kernel size 3 × 3, stride 2, same padding, and output channels are equal to 2 times basic units of the residual learning. 

The three convolutional layers are CConv1, CConv2, and CConv3. The filter sizes of the CConv1, CConv2, and CConv3 are 512, 256, and 128 respectively. The convolutional layers CConv1, CConv2, and CConv3 respectively contain six convolutional operations. The convolutional parameters of the first convolutional operation are the convolutional kernel size 1 × 1, and the number of output channels is equal to the filter size. The convolutional parameters of the second convolutional operation are the convolutional kernel size 3 × 3, and the number of output channels is equal to 2 times the filter size. The convolutional parameters of the third convolutional operation are the convolutional kernel size 1 × 1, and the number of output channels is equal to the filter size. The convolutional parameters of the fourth convolutional operation are the convolutional kernel size 3 × 3, and the number of output channels is equal to 2 times the filter size. The convolutional parameters of the fifth convolutional operation are the convolutional kernel size 1 × 1, and the number of output channels is equal to the filter size. The convolutional parameters of the sixth convolutional operation are the convolutional kernel size 3 × 3, and the number of output channels is equal to 2 times the filter size. The upsampling layers Upsample1 and Upsample2 sample the input feature maps and the input feature maps of the concat layer into the same size. 

**Figure 13:**
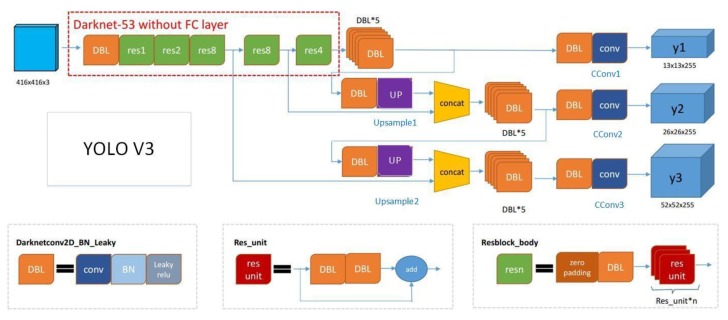
YOLO V3 network structure.

**Figure 14:**
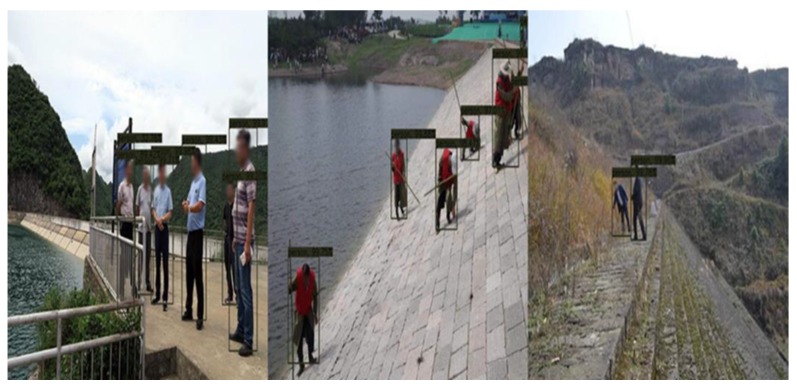
People detection results.

**Figure 15:**
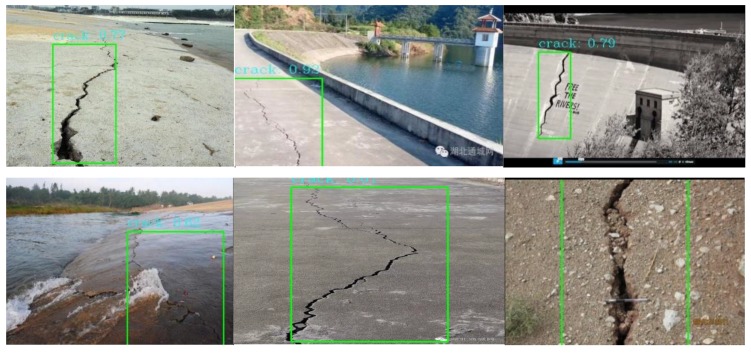
Dam crack detection results.

The network structure is shown in Figure 13. This method can do the detection job at 10 Hz with less than 20% CPU occupying. Some results are shown in Figure 14 and Figure 15.

## 8. Conclusions

In this paper, a general robot system is proposed for dam surveillance. The robot is connected to cloud servers and terminal users by mobile internet and IoT network. Like the robot itself, this paper introduces mechanics layout, sensor selection, and the navigation method. A simple controller and a wheel odometry calculation are proposed and achieve good performance. A two-node EKF structure localization framework is proposed to solve localization problem, which fuses LiDAR SLAM, wheel odometry, IMU, and GNSS signals. For unexpected circumstances, a remote controller based on real-time video streaming is deployed as an emergency supplement. To make the whole system able to work all-time, a control state machine is also introduced. A YOLO v3 network is trained and deployed to detect dam crack and people around. From the practical experiments, this system can work well and is capable of the surveillance job for dams. Afterward, we will pay more attention to specific dams’ surveillance jobs, such as intrusion detection and dam deformation detection. We believe robots will greatly improve the work efficiency for water conservancy in the future.
